# Effects of an Interdisciplinary Integrative Oncology Group-Based Program to Strengthen Resilience and Improve Quality of Life in Cancer Patients: Results of a Prospective Longitudinal Single-Center Study

**DOI:** 10.1177/15347354221081770

**Published:** 2022-02-28

**Authors:** Burcu Babadağ Savaş, Bettina Märtens, Holger Cramer, Petra Voiss, Julia Longolius, Axel Weiser, Yvonne Ziert, Hans Christiansen, Diana Steinmann

**Affiliations:** 1 Department of Radiation Therapy and Special Oncology, Medical School Hannover, Hannover, Germany; 2 University of Duisburg-Essen, Essen, Germany; 3 Department of Organisation, Innovation and Quality of Management, Medical School Hannover, Hannover, Germany; 4 Institute of Biometrics, Medical School Hannover, Hannover, Germany

**Keywords:** life of quality, resilience, integrative, complementary medicine, anxiety and depression, naturopathy, care, oncology, radiotherapy

## Abstract

**Background:**

Patients with cancer receiving oncological treatment often suffer from a reduced quality of life (QoL) and resilience.

**Objectives:**

The aim of this study was to evaluate the effect of an interdisciplinary integrative oncology group-based program on resilience and quality of life in patients with cancer during or after conventional oncological therapy.

**Methods:**

This prospective longitudinal single-center study evaluated the resilience (Resilience Scale), quality of life (EORTC-QLQ C30), anxiety, depression (Hospital Anxiety and Depression Scale), and distress levels (Distress Thermometer) of 60 patients with cancer who participated in a 10-week interdisciplinary integrative oncology group-based program during or after cancer treatment in outpatient clinics. An average of 12 (range 11-13) patients participated in each 10-week group. The program included recommendations for diet, stress management, relaxation, and exercise, as well as naturopathic self-help strategies and psychosocial support.

**Results:**

There were slight increases in global quality of life scores (week 0: 58.05 ± 20.05 vs week 10: 63.13 ± 18.51, n = 59, *P* = .063, *d* = −.25) and resilience scores (week 0: 63.50 ± 13.14 vs week 10: 66.15 ± 10.17, n = 52, *P* = .222, *d* = −.17) after the group program compared to before; however, these changes were not statistically significant and had small effect sizes. Patients with at least moderate anxiety symptoms (*P* = .022, *d* = .42) and low resilience (*P* = .006, *d* = −.54) benefited most from the program. The patients reported no relevant side effects or adverse events from the program.

**Conclusions:**

No significant effects on global quality of life or resilience were found in the general sample; notably, patients with anxiety and low initial resilience benefited the most from the program.

## Introduction

Patients with cancer may suffer from adverse effects, such as fatigue, pain, appetite loss and stress, during and after oncological therapies.^1^ Oncological diseases and these adverse effects often lead to depression, anxiety, distress, and hence impaired quality of life and weakened resilience.^2,3^ Resilience refers to the maintenance or rapid restoration of mental health during or after stressful life circumstances. Cancer diseases and adverse effects of oncological treatments are risk factors for stress-related mental illnesses.^3^ Patients with good resilience may cope with the disease and adverse effects of treatments, maintain a good psychological state and have a better quality of life.^4,5^ Resilience is the ability to adapt to stressors and is increasingly being recognized as dynamic and trainable.^3^ Supportive care during or after treatment often makes a positive contribution to patients’ health, resilience, quality of life, and well-being.^6^ It is important for patients with cancer to maintain their physical and mental health to prevent a reduction in their quality of life. Prior studies have demonstrated the importance of including cognitive behavioral therapy components and integrative medicine approaches in cancer care to maintain healthy lifestyle behaviors, improve their quality of life and reduce the side effects of cancer treatment.^7-9^

The German breast cancer guidelines recommend standardized interventions such as mindfulness-based stress management and exercise programs to improve the quality of life, coping strategies, and performance and fatigue.^10^ In addition, the German guidelines regarding supportive therapy in oncology recommend several integrative medicine self-help strategies (eg, cryotherapy or probiotics), whose effectiveness has been proven.^11^ United States (U.S.) guidelines include comparable recommendations: the clinical practice guidelines by the Society for Integrative Oncology, endorsed by ASCO,^1^ recommend mind-body therapies such as meditation, relaxation, yoga, massage, acupuncture, and music therapy as supportive treatment strategies effective for improving the quality of life during and after the treatment of patients with breast cancer.^12^

In particular, various integrative group-based programs (mindfulness-based cognitive therapy, self-empowerment programs, comprehensive treatment programs, etc.) have been shown to increase patients’ coping strategies with cancer and reduce distress, anxiety, and depression.^13-15^ Additionally, group-based integrative practices strengthen self-care in cancer patients and thereby increase their quality of life.^16,17^ A potential pathway for this effect is via integrative medicine treatment groups that make a positive contribution to the self-care process of the individual by fostering relationships among patients suffering from similar impairments such as fatigue, menopausal symptoms, sleep disorders, and sharing pragmatic experiences.^18,19^ Moreover, mutual peer support during cancer treatment makes a particularly important contribution to the process of emotional relaxation, disease acceptance and coping strategies. Integrative medicine group programs have also been shown to increase positive health behaviors and resilience.^3,20^ In group programs, patients take responsibility for promoting these pragmatic experiences and coping strategies to other patients.^21^ Therefore, it is important to integrate group-based peer support applications into the treatment and care process of patients with cancer.^21^

As seen in the literature,^16,20,22^ integrative medicine programs generally include several approaches available for patients with cancer; however, there are certain barriers to applying these approaches as routine practices in the hospital.^21^ These barriers include a lack of knowledge of health professionals about integrative or complementary approaches,^23^ generally inadequate information sharing of patients with the health care team about these approaches,^24^ or not allocating sufficient budgets for these practices in hospitals.^25^ In addition, Hinz et al conducted a study in 5 cancer centers in Germany and found that patients with cancer treated in outpatient clinics had a lower quality of life than patients treated in inpatient clinics. Additionally, they suggested that health care providers should offer their target-oriented psychosociological services to outpatients as well.^26^ Due to the heterogeneity of studies involving group-based integrative interventions in the literature and the limited number of supportive approaches, especially for outpatients in clinical practice, this study may guide further studies and clinical practice in integrative oncology. For this reason, an interdisciplinary integrative oncology group-based program in adult patients with cancer who receive outpatient services during or after conventional oncological therapy may have a positive effect on quality of life and resilience.

The objectives of this study were:

Primary objectives

- to compare the effects of an interdisciplinary integrative oncology group-based program on resilience and global quality of life level of patients with cancer at weeks 0 and 10.

Secondary objectives

- to compare the effects of an interdisciplinary integrative oncology group-based program on functional and symptom scales, anxiety, depression, and distress level of patients with cancer at weeks 0 and 10.
- to identify the risk groups that benefit most from the group program with subgroup analysis.

## Materials and Methods

### Study Design

This study was a prospective longitudinal, single-center study that evaluated the resilience, depression, anxiety, distress levels, and quality of life of patients who participated in the group program at the Medical School Hannover (MHH) in the Department of Radiotherapy and Special Oncology, Germany.

### Participants

Patients with cancer interested in group program contacted us, and a total of 110 patients were invited to participate in the group program by specialist physicians or nurses on naturopathy. Sixty of these patients agreed to participate in the group program and fill out the relevant questionnaires.

In summary, a total of 60 patients with cancer who met the inclusion criteria participated in the study between January 2019 and November 2020. The inclusion criteria of the study were cancer patients (during or after cancer therapy, aged ≥18 years, with adequate compliance and understanding of the German language, Karnofsky index ≥70) who had been treated for cancer currently or in the past in the outpatient department of radiotherapy or other outpatient departments of the MHH. Patients with neurological or serious mental illnesses were excluded. The group program was financially supported by the foundations Rut-und Klaus-Bahlsen-Stiftung and Förderstiftung MHH plus.

### Interdisciplinary Integrative Oncology Group-Based Program

The interdisciplinary integrative oncology group-based program has been applied in a university hospital department of radiotherapy since October 2018, and it is funded by Rut- und Klaus-Bahlsen-Stiftung and Förderstiftung MHH plus. This program is open to all adult patients with cancer during or after cancer therapy (including all cancer therapies) who have been treated currently or in the past in the outpatient departments of MHH. The program is still running and will be financially supported by the founders at least until 2025. The group program is announced through posters, brochures, business cards, websites, or naturopathic consultations conducted by physicians and nurses. Patients interested in the group program can reach the relevant department via e-mail or telephone, and the first appointment is made by the patients. The patients are counseled on integrative approaches and assessed by specialist physicians or nurses of naturopathy to determine whether they are suitable for group program attendance. The detailed information about the group program that we evaluated in this research and carried out between January 2019 and November 2020 is as follows:

The group program requires a total of 50 hours, comprising 5 hours on 1 day per week for 10 weeks. The program was integrated into standard therapy. The group program included evidence-based integrative medicine approaches, as detailed in Table 1.^10,11,12,27^ An average of 12 (11-13) patients participated in each 10-week group. There was no randomization in the creation of these groups; rather, a list was created according to the order of patient application. In creating the list, only the patients’ individual situations, such as other important appointments, planned holidays or rehabilitation treatments, were considered. The group program was carried out by an interdisciplinary team consisting of physicians, psychologists, nurses, physiotherapists, social scientists, and trainers (yoga, music, dance etc.), all of whom had training or expertise in integrative medicine. A physician leading the integrative oncology project started the group with a morning program consisting of exercise, meditation, and a group discussion about the previous week’s experiences and stressful situations with the aim of defining new directions and adopting new perspectives (neurocognitive restructuring). Other physicians, psychologists, nurses, and therapists conducted interactive sessions on yoga, dance therapy, Qi Gong, music therapy, etc.

**Table 1.** Content of the Interdisciplinary Integrative Oncology Group-Based Program.

Integrative oncology group program		Hours
Dietary recommendations (Mediterranean diet “whole foods,” dietary supplements, nutrition, and diet)		4
Exercise (morning exercise, yoga, Feldenkrais, dance therapy, Qi-gong, walking)		15
Relaxation (guided and sound meditations, creative therapy)		10
Stress management (mindfulness-based-stress-reduction-MBSR therapies, working with thoughts and feelings, meridian energy therapies (METs), partnership program “side by side” of the psycho-oncology of the MHH, music therapy, forest bathing)		6
Learning naturopathic self-help strategies (eg, manual therapies, aromatherapy, emotional freedom techniques (EFT) and tapping therapy, laughter yoga, homeopathy, breathing therapy)		5
Psychosocial support through group exchange		10
Content: 10 wk, 1×/wk, 9 am to 4 pm, including a 2-h break	Total	50

### Assessment and Measurements

Patients were evaluated by the questionnaires detailed below just before the start of the 10-week group program (week 0) and after the group program ended (week 10).

### Descriptive Characteristics Identification Form

The form was prepared according to recent studies.^16,20,22^ It consisted of 24 items, with the first 7 items related to sociodemographic data and the remaining items related to illness status, satisfaction, expectations, or difficulties during the group program.

### EORTC QLQ-C30 (Quality of Life Questionnaire)

The European Organization for Research and Treatment of Cancer (EORTC) Quality of Life Questionnaire (QLQ)-core (C-30) is comprised of multi-item scales and single-item measures. These include a *global quality of life* (QoL), 5 functional and 9 symptom scales. *Functional scales* include physical functioning (PF), role functioning (RF), emotional functioning (EF), cognitive functioning (CF), and social functioning (SF).^28^ *Symptom scales* include fatigue, pain, dyspnea, insomnia, constipation, diarrhea, appetite loss, financial difficulties, and nausea and vomiting.^28^ For functional scales and global QoL, higher scores represent higher functioning/QoL. For the symptom scales, higher scores indicate more severe symptoms.^28^ Each item is scored from 1 to 4 (“not at all”: 1; “a little”: 2; “quite slightly”: 3; “very much”: 4). As an exception, global QoL is scored from 1 (“very poor”) to 7 (“excellent”). The German validity and reliability of the scale have been established. The reliability coefficients (Cronbach’s alpha) for the functional scales were .80, and for the symptom scales, they were .63.^28-30^

### Resilience Scale (RS-13)

The German version of the Resilience scale consists of 13 items scored from 1 to 7, with higher scores indicating greater resilience. A score of 13 to 66 points indicates low resilience, a score of 67 to 72 points indicates medium resilience, and a score of 73 to 91 points indicates high resilience. The internal consistency of the scale is α = .90.^31^

### Hospital Anxiety and Depression Scale (HADS-D)

The German version of the HADS is used to evaluate anxiety and depression in patients with physical illnesses. The HADS-D consists of 14 items in total and has 2 subscales (anxiety and depression). Each item is scored between 0 and 3, with higher scores indicating more severe distress. Patients can be categorized based on their individual sum scores: noncase (0-7), borderline case (8-10), and definite case (11 and above). To identify patients with at least moderate symptoms of anxiety and depression, we used a cut-off score of >8.^32,33^

### Distress Thermometer

The distress thermometer is used to evaluate distress in oncology patients.^34,35^ It is rated between 0 and 10 as (0 = I have no distress and 10 = I have extreme distress). According to the National Comprehensive Cancer Network Distress Management Guidelines^34^ and recommendations by the authors of the German version,^35^ a score of 5 or greater indicates a distress level requiring patient support.^32,34,35^

### Statistical Analysis

Statistical analyses were performed using the Statistical Package for Social Sciences (SPSS; version 27). To describe the data (eg, patient characteristics, psychometric scales), standard univariate statistical analyses were applied. Categorical variables are shown as absolute and relative frequencies. Continuous variables are shown as the mean and standard deviation. For all scales, data distributions were evaluated by Shapiro–Wilk’s test. For normally distributed data, paired *t*-tests were used to compare the mean scores of the 4 questionnaires between week 0 and week 10. Cohen’s *d* effect sizes were calculated to express the mean score differences of patients for all scales between week 0 and week 10. Effect sizes were defined as *|d|* ≥ .2 small effect, *|d|* **≥** .5 medium effect, and *|d|* **≥** .8 large effect.^36^ For nonnormally distributed data, the Wilcoxon test was used.

The outcomes were further analyzed based on specific subgroups. First, patients were grouped based on the specific cut-offs for HADS (noncase, borderline, definite case, and >8 symptoms rated at least moderate), resilience (13-66, low resilience; 67-72, medium resilience, 73-91, high resilience), and distress (≥5, distress level requiring patient support), and changes in these subgroups were analyzed separately by Wilcoxon or McNemar tests.

SPSS, by default, conducts analyses by dropping cases for which there are missing values, so the sample sizes may differ in the statistical analyses. Inferential statistics are used in a descriptive manner. Thus, neither global nor local significance levels were determined, and no adjustment for multiplicity was applied. However, *P* values of .05 were considered statistically significant.

### Ethical Considerations

Ethics committee approval was obtained from the MHH Ethics Committee (approval number: 8204_BO_S_2018). Informed consent forms were obtained from all patients.

## Results

### Patient Characteristics (Sociodemographic and Medical)

Most (86.7%) of the patients were between 46 and 70 years old, female (95.0%), and married (53.3%). Most patients (71.7%) were followed up for breast cancer and had already completed at least 1 cancer treatment (86.7%). More than half of the patients (56.7%) received cancer treatment during the group program, and among these treatments, hormone therapy was the most frequent (33.3%). Only a minority of the patients (16.7%) were scheduled for further cancer treatment. The patient characteristics are shown in Table 2.

**Table 2.** Patient Characteristics (Socio-Demographic and Medical) (N = 60).

	n	%
Gender		
Female	57	95.0
Male	3	5.0
Age		
30-45 y	1	1.7
46-70 y	52	86.7
>70 y	7	11.7
Marital status		
Single	13	22.4
In a relationship	9	15.5
Married	32	55.2
Widowed	4	6.9
Education level		
Main elementary school	4	6.9
Secondary school	15	25.9
College/University education	8	13.8
General or subject-specific higher education	31	53.4
Cancer diagnosis		
Breast	43	71.7
Gynecologic (ovaries, uterus, cervix, or other)	9	15.0
Prostate	3	5.0
Lymphoma	3	5.0
Pancreatic	1	1.7
Brain	1	1.7
Completed cancer treatment before the group program*		
Surgical	45	75.0
Radiotherapy	43	71.7
Chemotherapy	28	46.7
Hormone therapy	17	28.3
Other treatments**	6	10.0
Ongoing cancer treatment during the group program*		
Hormone therapy	20	33.3
Chemotherapy	9	15.0
Other treatments**	7	11.7
Radiotherapy	3	5.0
Surgical	2	3.3

Abbreviation: n = numbers of patients.

* More than 1 option could be marked by the patient in the questionnaire.

** Immune therapy, interferon treatment etc.

Almost half of the patients (43.3%) who participated in the group program had learned about it during consultation with an integrative oncology physician. In our institution, a physician offers consultations by responding to integrative and naturopathic questions during cancer patient therapies. The patients were particularly interested in nutrition (78.3%) and mindfulness-based stress reduction (MBSR) (70.0%) before they joined the group program.

### Comparison of EORTC QLQ C-30 Scores at Weeks 0 and 10

A comparison of the pre- and post-integrative oncology group program EORTC-QLQ-C30 mean scores is shown in Table 3. An increase in global quality of life and functional scales and a decrease in symptom scales were observed after the group program compared to before; however, these changes were not statistically significant (*P* > .05). The change in global quality of life (*P* = .063, *d* = −.25) and social functioning scores (*P* = .052, *d* = −.26) had small effect sizes.

**Table 3.** Comparison of EORTC-QLQ-C30 Mean Scores at Week 0 and 10.

	Week 0	Week 10			
Scores	M ± SD	M ± SD	n	*P*-value*	Effect size (Cohen’s *d*)
Global QoL	58.05 ± 20.05	63.13 ± 18.51	59	.063	−.25
Functional scales					
Physical functioning	74.94 ± 20.22	76.20 ± 20.90	58	.571	−.08
Role functioning	56.60 ± 28.77	60.63 ± 28.90	58	.237	−.16
Emotional functioning	55.35 ± 17.79	55.95 ± 23.76	56	.834	−.03
Cognitive functioning	58.75 ± 24.63	60.45 ± 22.08	59	.536	−.08
Social functioning	59.64 ± 28.51	67.54 ± 30.11	57	.052	−.26
Symptom scales/items					
Fatigue	52.57 ± 27.42	50.00 ± 27.54	56	.443	.10
Nausea and vomiting	9.60 ± 17.00	4.80 ± 12.39	59	**.037**	.28
Pain	33.62 ± 30.66	34.19 ± 33.54	58	.888	−.02
Dyspnea	38.88 ± 34.26	37.22 ± 36.35	60	.678	.05
Insomnia	58.33 ± 36.63	47.22 ± 34.33	60	**.013**	.33
Appetite loss	10.00 ± 21.52	11.11 ± 22.68	60	.698	−.05
Constipation	15.81 ± 29.26	11.86 ± 24.57	59	.180	.18
Diarrhea	20.68 ± 31.73	18.39 ± 29.40	58	.532	.08
Financial difficulties	27.58 ± 32.52	22.41 ± 32.07	58	.172	.18

Abbreviations: QoL, Quality of Life; n, numbers of patients; M, mean; SD, Standard deviation.

* Paired sample *t* test; bold *P*-values indicate significant differences between time points (*P* < .05).

It was found that nausea-vomiting (*P* = .037, *d* = .28) and insomnia (*P* = .013, *d* = .33) scores significantly decreased after the integrative group program; however, these changes had a small effect size. The other scores had no effect with a relevant size (*P* > .05, *d* < .2) (Table 3).

### Comparison of HADS, Resilience, and Distress Thermometer Scores at Weeks 0 and 10

A comparison of the HADS, resilience, and distress thermometer mean scores between weeks 0 and 10 is shown in Table 4. There were no statistically significant differences in anxiety, depression, resilience, or distress thermometer mean scores before and after the integrative oncology program in the group as a whole (*P* > .05). Only the change in HADS anxiety score had a small effect size (*P* = .127, *d* = .21), and the other scores had no effect of a relevant size (*P* > .05, *d* < .2). However, in comparing the individual sum HADS scores—categorized as noncases, borderline cases and definite cases—anxiety was found to have decreased significantly after the group program (*P* < .05) (Table 4). Especially in patients with HADS anxiety scores above 8 (at least moderate symptoms), anxiety decreased significantly after the group program, and these changes had a small effect size (week 0: 9.33 ± 1.70 vs week 10: 7.96 ± 3.72, n = 33, *P* = .022, *d* = .42).

**Table 4.** Comparison of HADS, Resilience, Distress Thermometer Mean Scores at Weeks 0 and 10.

Scores	Week 0	Week 10				Week 0			Week 10					Week 0		Week 10	
HADS						Patient classes acc. Sum score (non-case = 0–7, borderline = 8–10, definite case ≥ 11, at least moderate symptoms > 8)											
	M ± SD	M ± SD	n	*P*-value*	Effect size (Cohen’s *d*)	0–7 (%)	8–10 (%)	≥11 (%)	0–7 (%)	8–10 (%)	≥11 (%)	n	*P*-value**	Cut off >8	Cut off >8	n	*P*-value***
HADS anxiety	7.73 ± 2.62	7.01 ± 3.67	53	0.127	0.21	21 (38.9)	26 (48.1)	7 (13.0)	39 (67.2)	9 (15.5)	10 (17.2)	53	**.038**	33 (61.1)	19 (32.8)	53	**.004**
HADS depression	5.96 ± 2.78	6.00 ± 3.11	58	0.930	−0.01	45 (76.3)	10 (16.9)	4 (6.8)	40 (67.8)	17 (28.8)	2 (3.4)	58	.499	14 (23.7)	19 (32.2)	58	.227
Resilience						Patient classes acc. Sum score (Low resilience: 13–66, medium resilience: 67–72, high resilience: 73–91)											
	M ± SD	M ± SD	n	*P*–value*	Effect size (Cohen’s *d*)	13–66 (%)	67–72 (%)	73–91 (%)	13–66 (%)	67–72 (%)	73–91 (%)	n	*P*-value**				
	63.50 ± 13.14	66.15 ± 10.17	52	0.222	−0.17	34 (60.7)	10 (17.9)	12 (21.4)	23 (41.1)	19 (33.9)	14 (25.0)	52	.265				
Distress thermometer						Patient classes acc. Sum score (≥5 signifies a distress level where the patient needs support)											
	M ± SD	M ± SD	n	*P*–value*	Effect size (Cohen’s *d*)	Cut off ≥ 5			Cut off ≥ 5			n	*P*–value***				
6.52 ± 2.08	6.57 ± 2.32	57	0.886	−0.02	45 (77.6%)			45 (76.3%)			57	1.000					

Abbreviations: IG, Integrative; HADS, Hospital Anxiety and Depression Scale; n, numbers of patients; M, mean; SD, Standard deviation.

* Paired sample *t* test, **Wilcoxon test, ***McNemar test; bold *P*-values indicate significant differences between time points (*P* < .05).

The mean resilience scores indicated that the majority of patients had low resilience before and after the integrative oncology program (Table 4). In the subgroup of patients with low initial resilience (sum scores: 13-66), resilience increased significantly after the group program, and these changes had a medium effect size (week 0: 55.93 ± 12.06 vs week 10: 64.20 ± 7.93, n = 30, *P* = .006, *d* = −.54).

The mean distress scores indicated that most patients needed support before and after the group program (Table 4). No significant difference or effect of relevant size was observed in the distress levels before and after the group program.

### Safety Aspects and Compliance

Patients reported no relevant side effects or adverse events. Patients reported difficulties during the group program, such as remembering their own childhoods, listening to other patients’ problems because they empathized, and hesitating to introduce themselves; they reported overcoming all of these difficulties with the support of the therapists. Only 6 patients participated in less than half of the 10-week group program due to illness, rehabilitation appointments, or personal reasons. There were no other records (eg, diaries, activity record lists, etc.) regarding the participants’ activities outside the program or their daily status. No other recommendation was given regarding their daily activities on other days or homework.

Patients attended an average of 8 of 10 sessions during the 10-week group program.

### Personal Statements of Patients After the End of the Integrative Group Program

After the program, most patients said they appreciated being able to participate in the group program. Some of these statements made by patients following the interdisciplinary integrative oncology group-based program are shown in Table 5. Not all patients gave feedback. The comments of the patients who gave feedback were positive, and statements were selected randomly from these comments.

**Table 5.** Quotes From Participants of the Interdisciplinary Integrative Oncology Group-Based Program.

Some of the statements made by patients after the integrative group program	“.. Today was a great day. I feel good. I can relax much better.”
	“. . .The group program offers a colorful bouquet of possibilities of what we can do for ourselves during and after cancer so that we feel well and can become and remain healthy. . .”
	“. . .The group program was for me personally a supportive help for orientation after the very exhausting treatment period. I thought the wide range of offerings in the group program was great.”
	“What I found especially good about the group program was that we saw many possibilities of what we could do to get through “daily life” better (more mindfully).... I thought the breakdown of the group day was great. . .The morning “meditation” and the discussion circles.... and then the many suggestions of what we can do, e.g. laughter yoga. I was able to laugh again after a long time and that moved me to tears.”
	“The program was so successful that I would heartily recommend it to any cancer patient or others with chronic illnesses. The initial skepticism wore off immediately. I still do the 20 minute morning exercises, yoga, meditations, walking in the forrest and tapping therapy now.... Not to mention all the lectures on nutrition, natural remedies, supplements, etc.”
	“The calm and pleasant voice of the therapist took me out of my everyday life and let me dive into another pleasant world.”
	“I didn’t want to go to laughter yoga at first. I had no expectations and was positively surprised.”
	“I will eat more consciously.”

## Discussion

This study investigated the effects of a 10-week interdisciplinary integrative oncology group-based program on resilience and improving quality of life during or after cancer treatment among 60 patients. Our results showed that there were slight increases in global quality of life and resilience scores after the group program compared to before; however, these changes were not statistically significant. Moreover, the change in the quality of life score and social functioning score had a small effect size, and the change in the resilience score had no effect of a relevant size. In the literature, group-based studies with patients with cancer have reported significant improvements in global health status, functional status, physical and emotional well-being, and personal strength, and decreases in cancer-related symptoms such as fatigue, sleep disorders and anxiety-depression.^15,17,37^ However, the effect size was not evaluated in these studies. Apart from these studies, other studies evaluating group-based interventions for patients with cancer actually calculated effect sizes. Accordingly, in the study of Haller et al, the effect of integrative mind-body practices during chemotherapy treatment in patients with breast cancer was examined. In their study, EORTC global quality of life (D = 9.5; 95% CI = [2.9|16.1]; *P* = .005), stress (D = −3.5; 95% CI = [−5|−2.1]; *P* = .000), anxiety (D = −3.8; 95% CI = [−4.9|−2.7]; *P* = .000), and depression (D = −3.9; 95% CI = [−4.9|−2.8]; *P* = .000) were also reduced.^38^ In another study, an 8-week interdisciplinary rehabilitation program after cancer treatment for patients with neck and breast cancer found moderate and large effects in reducing depression and stress and increasing quality of life (effect size: 0.6-0.9).^39^ Furthermore, the meta-analysis of Rense et al examined the effect of psychosocial interventions on the quality of life of patients with cancer (effect size: 0.65). It was found that the most important moderating variable was duration, with interventions of more than 12 weeks being significantly more effective than interventions of a shorter duration.^40^ In summary, it was shown in prior studies that group-based practices in cancer patients had a moderate to large effect on the quality of life, and group-based studies of 12 weeks and longer were more effective. Therefore, according to the results of our study, the small effect size of an integrative group program on the quality of life can potentially be explained by the fact that our study was limited only once a week and for a period of 10-weeks, with no further follow-up, or no extra daily activity plan to do at home.

In addition, 2 cancer-related symptoms, that is, *nausea/vomiting* and *insomnia*, were significantly decreased in the patients following the group program, but these changes had a small effect size. A potential explanation for the significant decrease in nausea and vomiting after the group program might be that most patients did not receive nausea-inducing treatments such as chemotherapy during the group program. Apart from this, it is difficult to say that the group program influenced the symptoms, since both nausea/vomiting and insomnia were evaluated with only 1 question on the quality-of-life scale, and we had no other data regarding these symptoms of the patients.

Although no significant differences were found, in the present study, anxiety and depression levels were slightly decreased, resilience levels were increased, and distress levels remained relatively high following the group program. Additionally, in these changes, only the anxiety scores had a small effect size. According to the established cut-offs, the patients with at least moderate anxiety symptoms (cut off >8) and low resilience (sum scores: 13-66) benefited most from the group program. Additionally, these changes had a medium effect size for low resilience scores and had a small effect for moderate anxiety scores. This is in line with a recent study that reported significantly decreased levels of perceived stress, anxiety, and depression in breast cancer patients undergoing chemotherapy after an integrative mind-body-medicine group program. Jeitler et al^37^ tested a mindfulness-based group program in cancer survivors by means of a cohort study design with a waiting-list group. They found that anxiety/depression levels decreased after the program and that anxiety levels were higher than depression at baseline, and the improvement in anxiety levels was more pronounced compared to that in depression levels. Several systematic reviews^41-43^ reported that the resilience levels of individuals with cancer or chronic diseases are low and that symptom management is adversely affected. Ludolph et al reviewed 22 studies regarding resilience-promoting interventions in patients with cancer and found that the interventions that achieved the most positive effects included those that used positive psychology, supportive group therapy and behavioral or mindfulness-based measures. They found that the effect size ranged from g = 0.33 to g = 1.45 and that these interventions should extend over more than 12 sessions whenever possible.^3^ Although the use of group-based interventions in this review was similar to our study, these reviewed studies were mostly based on positive psychology parallel to somatic treatment and differed from our study in these aspects. It is known that group programs such as acceptance and commitment group therapy, laughter therapy or mindfulness-based cognitive therapy enhance the quality of life while simultaneously increasing resilience and wellbeing.^14,44,45^ Additionally, patients with high resilience and a high quality of life were more likely to cope well with cancer and the treatment side effects.^4,5^ Furthermore, in the clinical practice guidelines on the evidence-based use of integrative therapies during and after breast cancer treatment, meditation, relaxation, massage, and yoga and stress management and music therapy were recommended to reduce anxiety/stress and depression/mood disorders.^12^ Similarly, in our study, a group program was presented to patients with cancer, mainly breast cancer, by an interdisciplinary team. As suggested in the clinical practice guidelines, the content of our group program included several elements, such as relaxation, coping with stress, exercise, yoga, etc. Additionally, it should be emphasized that stress during the first few months after diagnosis seems to have an unfavorable effect on the future quality of life (especially with regard to the physical symptoms) of breast cancer patients.^46,47^ Integrative medical group programs should already be implemented for patients during active treatment shortly after diagnosis to promote resilience.^3^ Therefore, it seems important to establish integrative medicine group programs that include evidence-based practices and to screen patients with cancer for the mentioned factors (low resilience, impaired quality of life, sociodemographic features, psychological impairment such as anxiety, distress, and depression).

There are certain limitations to the present study, including the longitudinal data analysis, the single center considered, the lack of an a priori sample size calculation and the absence of a control group. Due to the restrictions implemented during the COVID-19 pandemic period, we experienced decreases in the sample size and the number of organized group programs. We did not record the tumor stage or type of treatment stage (curative or palliative, etc.) or whether patients received negative news regarding their cancer stage during the group program. We did not assess the quantity and quality of practice sessions, and we did not record the participants’ activities outside of the program or their daily status.

This project provides the first hints at the effectiveness of an evidence-based integrative oncology group-based program conducted by an interdisciplinary team in cancer patients during or after conventional oncological therapy.

## Conclusion

We found no statistically significant effects of an interdisciplinary integrative oncology group-based program that included recommendations for diet, stress management, relaxation, and exercise, as well as naturopathic self-help strategies and psychosocial support through group exchange, to improve the quality of life and resilience. In addition, the change in the quality-of-life score had a small effect size, and the change in resilience score had no effect on the effect size after the group program compared to before. Further research is needed to confirm the preliminary findings of more pronounced effects in patients with low initial resilience and high symptom burdens. In addition, our results suggest that the quantity and quality of practice sessions, the participants’ activities outside of the group program or daily activities should be assessed.
